# Investigation and Modeling of the Electrical Conductivity of Graphene Nanoplatelets-Loaded Doped-Polypyrrole

**DOI:** 10.3390/polym13071034

**Published:** 2021-03-26

**Authors:** Oladipo Folorunso, Yskandar Hamam, Rotimi Sadiku, Suprakas Sinha Ray, Neeraj Kumar

**Affiliations:** 1 Department of Electrical Engineering, French South African Institute of Technology (F’SATI), Tshwane University of Technology, Pretoria 0001, South Africa; HamamA@tut.ac.za; 2 Centre for Nanostructures and Advanced Materials, DSI-CSIR Nanotechnology Innovation Centre, Council for Scientific and Industrial Research, Pretoria 0001, South Africa; rsuprakas@csir.co.za (S.S.R.); NKumar@csir.co.za (N.K.); 3 École Supérieure d’Ingénieurs en Électrotechnique et Électronique, Cité Descartes, 2 Boulevard Blaise Pascal, Noisy-le-Grand, 93160 Paris, France; 4 Department of Chemical, Institute of NanoEngineering Research (INER), Metallurgy and Material Engineering, Tshwane University of Technology, Pretoria 0001, South Africa; sadikur@tut.ac.za; 5 Department of Chemical Sciences, University of Johannesburg, Doornfontein, Johannesburg 2028, South Africa

**Keywords:** graphene, polypyrrole, electrical conductivity, models, hybrid, percolation

## Abstract

In this study, a hybrid of graphene nanoplatelets with a polypyrrole having 20 wt.% loading of carbon-black ($\mathrm{HGPPy}.{\mathrm{CB}}_{20\%}$), has been fabricated. The thermal stability, structural changes, morphology, and the electrical conductivity of the hybrids were investigated using thermogravimetric analyzer, differential scanning calorimeter, X-ray diffraction analyzer, scanning electron microscope, and laboratory electrical conductivity device. The morphology of the hybrid shows well dispersion of graphene nanoplatelets on the surface of the $\mathrm{PPy}.{\mathrm{CB}}_{20\%}$ and the transformation of the gravel-like $\mathrm{PPy}.{\mathrm{CB}}_{20\%}$ shape to compact spherical shape. Moreover, the hybrid’s electrical conductivity measurements showed percolation threshold at 0.15 wt.% of the graphene nanoplatelets content and the curve is non-linear. The electrical conductivity data were analyzed by comparing different existing models (Weber, Clingerman and Taherian). The results show that Taherian and Clingerman models, which consider the aspect ratio, roundness, wettability, filler electrical conductivity, surface interaction, and volume fractions, closely described the experimental data. From these results, it is evident that Taherian and Clingerman models can be modified for better prediction of the hybrids electrical conductivity measurements. In addition, this study shows that graphene nanoplatelets are essential and have a significant influence on the modification of $\mathrm{PPy}.{\mathrm{CB}}_{20\%}$ for energy storage applications.

## 1. Introduction

Polypyrrole (PPy) is a conducting polymer, which has been examined to be a semiconductor but possesses excellent electrochemical characteristics [1]. The very viable possibility of this very versatile polymer for energy storage requires adequate modification of its properties and the subsequent characterization of its electrical conductivity. The $\mathrm{PPy}.{\mathrm{CB}}_{20\%}$ is a good energy storage material due to its high electrical conductivity, high surface area, excellent chemical and thermal stability [2,3,4]. 

An allotrope of carbon that is excellent in energy storage and filler for composite materials, is graphene. Graphene (Gr) is excellent in surface area and electrical conductivity. Energy storage devices require materials that are electrically conductive and thermally stable for their proper performances. Porosity, mechanical and electrochemical stability contribute to the emphasis on the applications of Gr for energy storage. It is important to note that there are different types of graphene and the use of the proper nomenclature of the type of graphene materials employed in any study, would preserve the integrity of carbon science community. Fullerenes, carbon-nanotube, synthetic diamond, and n-layer graphene (n = single, few, and multi), are allotropes of carbon materials [5,6]. In this study, the type of graphene used is graphene nanoplates. Graphene nanoplatelets are multilayer graphene that can be produced by chemical, thermal, or mechanical exfoliation methods [7,8]. Graphene with layer number ranging between 2 and 5 [7], or probably 2 and 10 [8] layers, are usually referred to as few-layer graphene. Investigations conducted by Kumar and Lee [9] and Kumar et al. [10], showed that few-layer graphene can effectively modify the properties (electrical and mechanical) of vulcanized silicone rubber at room temperature, for piezo-electric actuator application. Consequently, graphene of any type has plethora advantages in reinforcing and enhancing the properties of polymers for applications in different areas. The extant of Gr in polymer composites supports the polymer electroactive content at the nanoscale. This effect will improve the specific surface area, electrochemical stability, and electrical conductivity of the final composite material [11,12]. The use of multi-fillers can find advantages in the improvement of shape memory polymers. For example, Lu and Huang [13], investigated the synergetic effects of carbon-nanotube and boron-nitride: the results of the investigation showed that carbon-nanotube has the tendency to enhance the thermal conductivity and the infrared-light absorption properties of shape memory polymer. The recovery of the infrared-light induced shape and the heat transfer in the shape memory polymer, can be potently facilitated by the boron-nitride. In addition, the hybrid of carbon-fiber and carbon-nanotube has been reported as a viable method to enhance the electrical conductivity of shape memory polymer [14].

Various reports have shown that the composite of Gr-PPy is an excellent means of producing supercapacitors and batteries for large scale and automobile energy storage application [15,16,17,18,19,20,21]. Zuo et al. [21] carried out an experimental investigation on the sulfonated Gr-PPy composite by using a one-step electrochemical preparation method. The fabricated electrode exhibited high specific capacitance and good electrochemical stability. Shu et al. [20], constructed a Gr-PPy electrode by employing electro-polymerization method. It was stated by Shu et al. [20] that the Gr-PPy electrode is suitable for flexible supercapacitor applications. de Olivera et al. [17] reported an in situ oxidative polymerization method to fabricate Gr-PPy electrode. The polymerization method enhanced the blending of the PPy with Gr, which resulted in high conductivity and excellent storage capability to the composite’s high conductivity and excellent storage capability. Ding et al. [18] explored the diameter-controlled method to produce Gr-PPy supercapacitor *via* a wet-spinning route. Good mechanical flexibility and high capacitance are advantages that were, reported for the Gr-PPy electrode produced. Ghosh et al. [11] used the in situ polymerization to prepare Gr-PPy electrode, yielded a 6480 W/kg power density. The electrode’s very high-power density can be attributed to the synergetic interaction of Gr and PPy, which is a function of the polymerization method. In a report by Bora et al. [16], a 90% capacity retention after 500 cycles was recorded for Gr-PPy electrode, prepared via interfacial polymerization method. However, the composite of polymer with other material can be easily produced by solvent blending (SSB) process. The SSB process has the advantages of simplicity, homogenous dispersion, and enhance properties (electrical, mechanical, thermal, and chemical) of the hybrid materials without deterioration [22,23,24].

It is evident that there are numerous investigations on the performances of Gr-PPy for energy storage, but there is the need to investigate further the ability of Gr to improve the properties of PPy composite containing other conductive fillers. Therefore, this study presents the SSB process preparation of the hybrid of Gr and $\mathrm{PPy}.{\mathrm{CB}}_{20\%}$ and their electrical conductivity models. In addition, there is a limited number of analytical investigations of this electrode, along with experimental measurements. The electrical conductivity of polymer-composites depends on many factors, which may not be fully described or understood via experimentation but can be, explained by some mathematical equations. Among others, some of the factors that influence the electrical conductivity of polymer-composites, are the orientation angle, surface interaction, aspect ratio, volume fraction, filler, and the polymer conductivity [25,26]. Upon the inclusion of filler into a polymer and at lower weight fraction, the composite’s conductivity would be equal to the conductivity of the polymer. 

Nevertheless, as the weight fraction of the filler is increased, the point is reached where the conductivity experiences a continuous linear increase until a saturation point is reached where the weight fraction no longer affects the conductivity. This condition is, usually described by the percolation theory and the point from which the composite gains continuous conductivity is called the percolation threshold. Clingerman and Taherian equations, are percolation theory models, which were developed based on statistical, geometrical, and thermodynamic formulation to predict the electrical conductivity of polymer-composites. Basically, these models obey the percolation theory of electrical conductivity with respect to volume fraction [27,28,29]. This study focused on the investigations of some existing models, needed to describe the electrical conductivity of $\mathrm{HGPPy}.{\mathrm{CB}}_{20\%}$.

The objective of this study is to investigate the electrical conductivity of the hybrid of graphene nanoplatelets with $\mathrm{PPy}.{\mathrm{CB}}_{20\%}$, in relation to its other properties. The procedures employed are: (i) experimentally study morphology, thermal stability, and structural changes of $\mathrm{HGPPy}.{\mathrm{CB}}_{20\%}$; (ii) experimentally measure the electrical conductivity of $\mathrm{HGPPy}.{\mathrm{CB}}_{20\%}$; (iii) analytically trace the trajectory of the hybrid’s electrical conductivity curve and develop mathematical equations for the observed curve. Weber [30], Clingerman [29] and Taherian [31] models were used to perform the analysis of the electrical conductivity measurements. The models were able to describe the experimental data, and from the results, subsequent equations were developed, which can be applied to closely predict the electrical conductivity of similar hybrids. However, the Clingerman and Taherian models will be further modified to generate new models in a successive study for improved electrical conductivity measurements of polymer composites. For further insights into this endeavor, refer to [32,33]. Critical observations of the experimental results show clear indications that $\mathrm{HGPPy}.{\mathrm{CB}}_{20\%}$ is a viable product for energy storage.

## 2. Electrical Conductivity Models

The analytical models for polymer-composites electrical conductivity prediction follows the empirical rule of mixture, percolation theory, and/or mathematical models. Since the electrical conductivity of a composite depends, largely on the volume fraction of filler content, it is reasonable that these composites’ behaviors be analyzed by varying the filler contents, in conjunction with other parameters by using some sets of mathematical equations. Percolation theory is most useful for predicting the electrical conductivity of composites due to their correlation in phase transitions of experimental results of polymer-composites. The point at which the composite forms a complete network for charges to transit from one end of the material to another without any resistance is called percolation threshold. A composite that is obtained with less percolation threshold is more economical since it will require a little amount of fillers to produce the materials needed for energy storage, sensors, and electronic devices. Filler size, surface energy, interfacial and tunneling effects, are other factors, which contribute to the electrical conductivity of polymer-composites. In general, the electrical conductivity of polymer-composites is based on two types of resistance, which are: intrinsic and tunneling resistances. For some fillers, intrinsic resistance is approximately equal to zero, then, tunneling or contact resistance, is another important factor which affects the electrical conductivity of polymer-composites. Tunneling resistance occurs due to filler-filler and matrix-filler interactions. The higher the thickness of the matrix between filler-filler, the greater the tunneling resistance, and the lower the electrical conductivity of the composite [34,35]. In addition, filler shape/size, matrix-crystallinity, and matrix-filler dispersibility [36], are among the various factors, which affect the electrical conductivity of polymer-composite [27].

Kirkpatrick [37] proposed the first statistical percolation theory, often referred to as the power-law equation. The power-law equation is as presented in Equation (1). The equation is effective, but its accuracy is questionable [28,38].

(1) $$\sigma_{c}=\;\sigma_{p}{\left(V-V_{c}\right)}^{k}$$

where $\sigma_{c}$ is the composite electrical conductivity, $\sigma_{p}$ is the matrix conductivity, V and $V_{c}$ are the volume fractions of the filler and the percolation threshold, k is the critical exponent. 

Weber model [30] focused on the filler volume fraction, dimensions, orientation, and aspect ratio. The model was developed to study the electrical conductivity of nickel-coated graphite, loaded on polypropylene. The composite conductivity is assumed to be proportional to the filler conductivity $\left(\sigma_{f}\right)$, as shown in Equation (2).

(2) $$\sigma_{c}\propto \;\sigma_{f}$$

The resulting electrical conductivity equation of the Weber model is given in Equation (3).

(3) $$\sigma_{c}=\;\frac{4\Phi_{p}\sigma_{f}d_{c}l\mathrm{co}s^{2}\theta }{\pi d^{2}X}$$

where $\Phi_{p}=\beta \Phi$, β is the percolation function, Φ is the volume fraction of the filler, θ is the orientation angle, l and d are the filler length and diameter, $d_{c}$ is the contact diameter, $\sigma_{f}$ is the filler conductivity, $X={\left(0.59+0.15m\right)}^{-1}$, and m is the contact number.

Clingerman [29] presented a general mixing rule equation, which is similar to the model of McCullough [39]. The model considered some factors, which account for the proper prediction of polymer-composite electrical conductivity. The base-ten logarithm model of Clingerman consisted of polymer electrical conductivity, $\sigma_{p}$, filler electrical conductivity, $\sigma_{f}$, volume fraction, ϕ, percolation threshold, $\phi_{c}$, critical exponent, t, aspect ratio, a, orientation angle, w, and surface interaction parameter, $\delta_{pf}$. The model is as presented in Equation (4).

(4) $$\log\left(\sigma \right)=\;\{\begin{array}{ll} \log\left(\sigma_{p}\right) & \emptyset \leq \emptyset_{c}\\ \log(\sigma_{p})+D\log(\sigma_{f}){\left(\emptyset -\emptyset_{c}\right)}^{t}+h\left(a\right)\cos\left(w_{pf}\right)-\gamma \delta_{pf} & \emptyset >\emptyset_{c} \end{array}$$

(5) $$t=\;\frac{U\emptyset_{c}}{{\left(\emptyset -\;\emptyset_{c}\right)}^{n}}$$

(6) $$h\left(a\right)=\;\{\begin{array}{ll} \left(\frac{a^{2}}{a^{2}-1}\left(1-0.5\left(A-\;\frac{1}{A}\right)\mathrm{In}\left(\frac{A+1}{A-1}\right)\right)\right) & for\;1\;<a\;<\infty \\ 1 & for\;a\;\to \infty \end{array}$$

(7) $$A=\sqrt{\left(\frac{a^{2}}{a^{2}-1}\right)}$$

The first term in Equation (4) accounts for the initial conductivity of the polymer, the second term accounts for the conductivity in relation to the volume fraction, the third accounts for the structure of the composite, the fourth term accounts for the surface energy experienced by the composite. Other parameters, such as: D, U, n, and γ are constants quantities. From the size of the Gr considered in this study, Equations (4)–(7) reduce to Equation (8).

(8) $$\log\left(\sigma \right)=\log\left(\sigma_{p}\right)+D\log\left(\sigma_{f}\right){\left(\emptyset -\;\emptyset_{c}\right)}^{t}+0.8256\cos\left(w_{pf}\right)-\gamma \delta_{pf}\;$$

Taherian [31] applied the Sigmoidal equation to predict the conductivity of polymer-composites by replacing the various constants, such as: a,b,c, and $x_{o}$ in Equation (9) with four factors considered to be the critical factors, which affect the electrical conductivity of polymer-composites. These four factors are: wettability of filler and polymer, filler aspect ratio, filler roundness, and filler conductivity. The following assumptions were made.

(9) $$f\left(x\right)=\;\frac{a}{c+\exp\left(-\frac{\left(x-x_{o}\right)}{b}\right)}$$

- Aspect ratio is concerned with either shifting the percolation threshold leftward or upward; therefore, the aspect ratio can substitute the constant a in the Sigmoidal equation.
- The filler roundness has an inverse relationship to the aspect ratio, as does the constant a to $x_{o}$; in this case, the filler roundness replaced the constant $x_{o}$.
- The influence of surface energy on the composite is proportional to the orientation of the fillers in the matrix. This effect has an inverse impact on the electrical conductivity, as does the constant  b. Therefore, the surface energy replaces the constant *b*.
- The volume fraction is taken as x.

The sigmoidal equation developed by Taherian is presented by Equation (10).

(10) $$\sigma_{c}=\;\frac{a{}^{\circ}\sigma_{f}}{c{}^{\circ}+\exp\left(-\frac{x-r}{\cos\left(\theta \right)}\right)}$$

where r is the roundness, $\sigma_{f}$ is the filler conductivity, cos(θ) is the polymer wettability, x is the volume fraction, and c° is a constant. The polymer wettability is, given in Equation (11).

(11) $$\cos\left(\theta \right)=\frac{\gamma_{s}-\;\gamma_{sL}}{\gamma_{L}},\gamma_{sL}=\;\gamma_{s}+\;\gamma_{L}-2{\left(\gamma_{s}\times \gamma_{L}\right)}^{\frac{1}{2}}$$

Herein, γ and θ are the surface energy of the polymer/filler and the wetting angle, the subscripts s and L represent the filler and matrix. For the polypyrrole and the carbon-materials considered in this study, surface energies of $36.18\;\mathrm{mJ}/m^{2}$ [40] and $24\;\mathrm{mJ}/m^{2}$ [31] were used to calculate the wetting angle; also used was a 0.995 graphene roundness [31]. By introducing a fifth term parameter, τ, into the Taherian model, Equation (12) is developed to predict the electrical conductivity of the $\mathrm{HGPPy}.{\mathrm{CB}}_{20\%}$ composite. The importance of the filler growth rate is to have control over the shape of the model.

(12) $$\sigma_{c}=\;\frac{a{}^{\circ}\sigma_{f}}{c{}^{\circ}+\exp-\tau \left(\frac{x-r}{\cos\left(\theta \right)}\right)}=\;\frac{a{}^{\circ}\sigma_{f}}{c{}^{\circ}+{\;\exp}^{-b{}^{\circ}\left(x-r\right)}}$$

where b°= τ/cosθ.

## 3. Materials

The graphene nanoplatelets and $\mathrm{PPy}.{\mathrm{CB}}_{20\%}$ (code no: 530573-25G) were supplied by Sigma Aldrich, South Africa. The description of the graphene nanoplatelets by the supplier has a surface area of between 50 and 80 m^2^/g, bulk density of 0.03–0.1 g/cm^3^, a diameter of 5 μm, an average thickness of 15 nm, and <1% oxygen content. The PPy has a 20 wt.% loading of carbon black. Deionized water was used for the experiment. 

The thermal analysis of the hybrids and the individual materials were carried out under nitrogen atmosphere using a TGA 5500 (TA instruments, New Castle, DE, USA). The average mass of the samples was 5 mg, at a heating rate of 10 °C/min. Further thermal analysis was carried out by a Q2000 differential scanning calorimeter (DSC, TA instruments, New Castle, DE, USA). An average mass of the samples was 3 mg at heating rate of 10 °C/min. 

The X-ray diffraction (XRD) structures of all the individual materials and the hybrids were recorded by using the X-ray generator (Xpert Pro X-Ray Diffractometer Panalytical, Eindhoven, The Netherlands) and CuKα radiation (λ=0.154 nm;I=40 mA, and V=45 kV). The X-ray diffractograms were obtained in the 2θ range between 5° and 90°, at a continuous scan step size of 0.0263°. 

The hybrids’ morphologies and the individual materials were studied by using a scanning electron microscope (SEM) equipment, Auriga workstation, produced by Carl Zeiss (Oberkochen, Germany) with an accelerating voltage of 3.0 kV at 1 μm. Each of the sample was prepared, and chromium-plated prior to imaging. 

## 4. Processing of Polymer Composites

The hybrid of Gr and $\mathrm{PPy}.{\mathrm{CB}}_{20\%}$ was, prepared by the SSB process. The mass of Gr to $\mathrm{PPy}.{\mathrm{CB}}_{20\%}$ was varied, thus: 1:19, 1:9, 3:17, 1:4, 1:3, 3:7, and 7:13; in which the resultant hybrids were coded: $\mathrm{HGPPy}.{\mathrm{CB}}_{20\%}$ 1:19, $\mathrm{HGPPy}.{\mathrm{CB}}_{20\%}$ 1:9, $\mathrm{HGPPy}.{\mathrm{CB}}_{20\%}$ 3:17, $\mathrm{HGPPy}.{\mathrm{CB}}_{20\%}$ 1:4, $\mathrm{HGPPy}.{\mathrm{CB}}_{20\%}$ 1:3, $\mathrm{HGPPy}.{\mathrm{CB}}_{20\%}$ 3:7, and $\mathrm{HGPPy}.{\mathrm{CB}}_{20\%}$ 7:13. In order to obtain the hybridized product, Gr was dispersed in deionized water by ultrasonication for 40 min. Second, the $\mathrm{PPy}.{\mathrm{CB}}_{20\%}$ was dispersed in 1:3 mixture of deionized water and ethanol. The product obtained in step 2 by a 1:3 mixture of deionized water and ethanol was added to the exfoliated graphene and ultrasonicated for 40 min. Furthermore, $\mathrm{HGPPy}.{\mathrm{CB}}_{20\%}$ product in step 3 was vigorously stirred for ~24 h at 300 rpm and 30 °C. Finally, the mixture was filtered and washed with ethanol/deionized water and dried in an oven for 24 h at 80 °C. 

### The Electrical Conductivity Measurement

**Figure 1:**
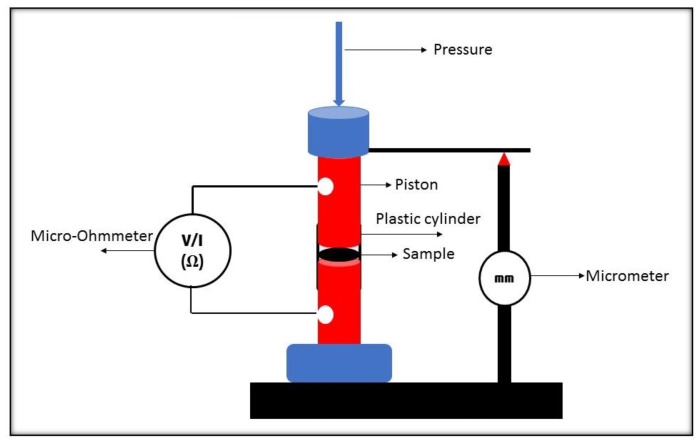
Laboratory electrical conductivity measurement setup.

The hybrid powders’ electrical conductivity measurements were performed using a developed laboratory measurement system, as shown in Figure 1 [41,42]. An Agilent 34420A, $7\frac{1}{2}$ digit nanovolt, micro-ohmmeter, was used to measure the powder samples’ electrical conductivity at room temperature. At a constant mass, each sample was loaded into an insulating cylinder of 0.89 cm inner diameter and compressed between two electrical conducting pistons. Since a constant pressure was applied to all the samples, the measured separating distance (ζ) between the upper and the lower pistons, by a micrometer, was constant. Several researchers [41,42,43,44] have measured powder samples’ electrical conductivity by using the present approach, employed in this study. The conduction was, measured in the Ohmic, therefore, the electrical conductivity was calculated using Equation (13).

(13) $$\sigma =\;\frac{\zeta }{RA}$$

where ζ is the thickness of the sample between the pistons, R is the measured electrical resistance, and A is the cross-sectional area of the pistons segment.

## 5. Results and Discussion

In this section, the results and discussion on the structural, thermal, and morphology characterizations of the hybrids are presented. More so, the laboratory determined electrical measurements of the hybrids are discussed. In addition, the comparisons of the experimental measurements are considered by using different electrical conductivity models.

### 5.1. Morphology Analysis of the Hybrids

**Figure 2:**
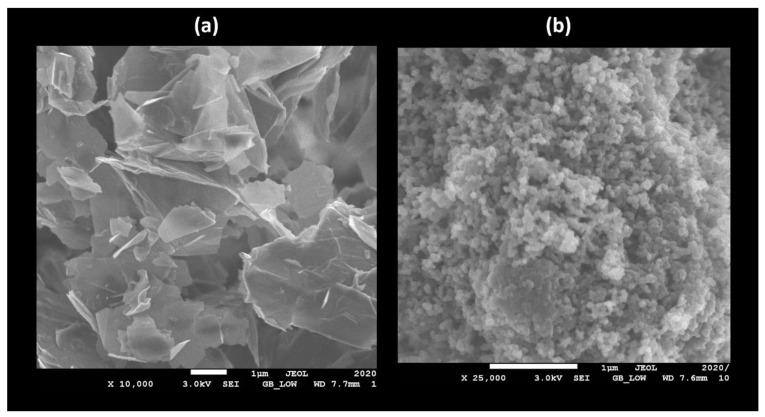
SEM graphs of (**a**) Gr and (**b**) $\mathrm{PPy}.{\mathrm{CB}}_{20\%}.$

The surface morphology of the $\mathrm{PPy}.{\mathrm{CB}}_{20\%}$ changes with Gr loading was performed by SEM. For a polymer-composite, the phase transformation of the morphology of the polymer with respect to nanomaterial has effects on the electrical conductivity of their hybrids. The SEM images of Gr and $\mathrm{PPy}.{\mathrm{CB}}_{20\%}$ are shown in Figure 2. The $\mathrm{PPy}.{\mathrm{CB}}_{20\%}$ shows a spherical gravel-like porous structure, which mounts on top of each other. From the analysis of the $\mathrm{PPy}.{\mathrm{CB}}_{20\%}$ morphology, $21,000\;nm^{2},\;57,850\;\mathrm{nm},$ and 90 nm are the surface area, diameter, and length of the polymer, respectively. The calculated Gr nanoplatelet average area, diameter, and length are: $235,000\;nm^{2},\;2.5\;\mu m,$ and 5 μm, respectively. It is evident from the calculated size of the Gr that it has a large aspect ratio that is required to promote contact with the $\mathrm{PPy}.{\mathrm{CB}}_{20\%}$. 

**Figure 3:**
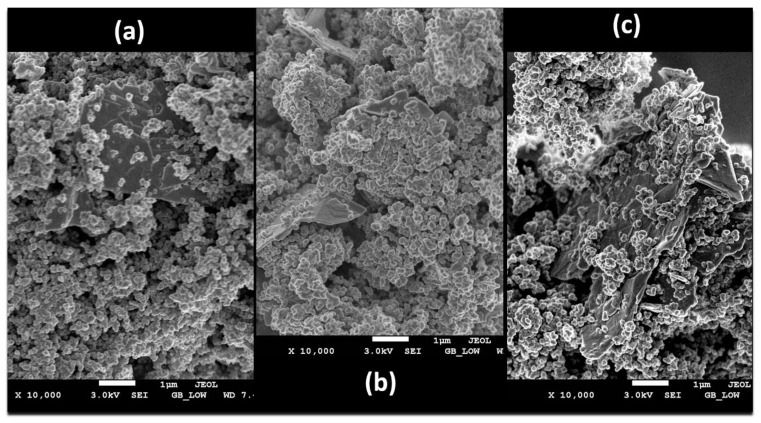
SEM graphs of (**a**) $\mathrm{HGPPy}.{\mathrm{CB}}_{20\%}$ 1:3 (**b**) $\mathrm{HGPPy}.{\mathrm{CB}}_{20\%}$ 3:7 (**c**) $\mathrm{HGPPy}.{\mathrm{CB}}_{20\%}$ 7:13.

More so, the surface morphologies of the hybrids of $\mathrm{HGPPy}.{\mathrm{CB}}_{20\%}$ are shown in Figure 3a–c. As seen in Figure 3a–c, the surface of $\mathrm{PPy}.{\mathrm{CB}}_{20\%}$ has been successfully modified by the Gr nanoplatelets. The $\mathrm{HGPPy}.{\mathrm{CB}}_{20\%}$ 1:3, shows homogenous and the well-dispersion of Gr on the surface of the $\mathrm{PPy}.{\mathrm{CB}}_{20\%}$, which resulted in a compact spherical morphology. Moreover, the weight fraction of the Gr determines to what extent the morphology of the polymer can be controlled, as shown in Figure 3b,c. The small quantity of agglomeration of $\mathrm{PPy}.{\mathrm{CB}}_{20\%}$, formed on the surface of the hybrids, proved that the hybrids formations are by *via* the π−π interactions and the van der Waals force [45,46]. The results show that the preparation method of the hybrid is appropriate in maintaining the integrity of the properties of the material and their homogenous dispersion.

### 5.2. Thermal Analysis of the Hybrids

PPy is a conducting polymer whose redox activities largely depends on temperature. The redox property of PPy can be improved with moderate temperature, but at high temperature, the material would be degraded. In addition, the moderate temperature has a positive effect on the electrical conductivity of the polymer and vice-versa [47]. Therefore, the thermal analysis of the $\mathrm{HGPPy}.{\mathrm{CB}}_{20\%}$ was carried to investigate an extent to how Gr can improve the thermal stability of the $\mathrm{PPy}.{\mathrm{CB}}_{20\%}$ and at what temperature the hybrids would still retain thermal integrity without degradation.

**Figure 4:**
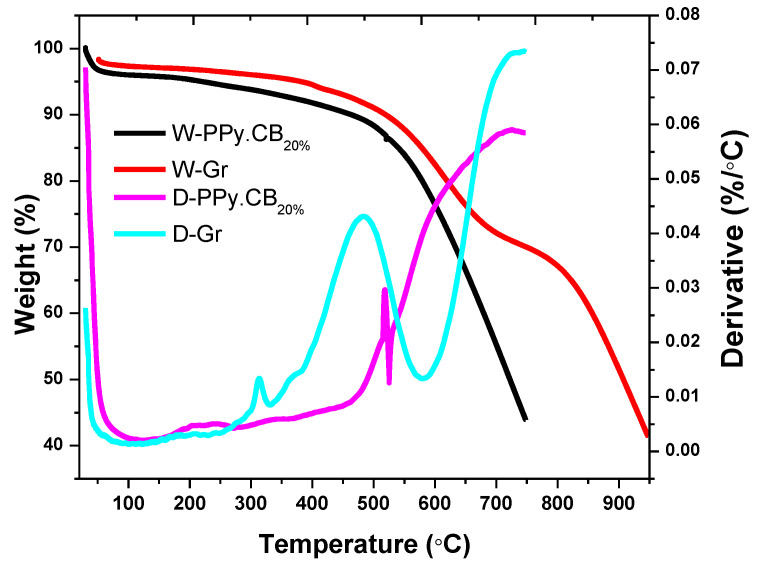
Temperature of analysis of Gr and $\mathrm{PPy}.{\mathrm{CB}}_{20\%}$. W-PPy.CB_20%_ and W-Gr are the percentage weight curves. D-PPy.CB_20%_ and D-Gr are the derivatives curves.

**Figure 5:**
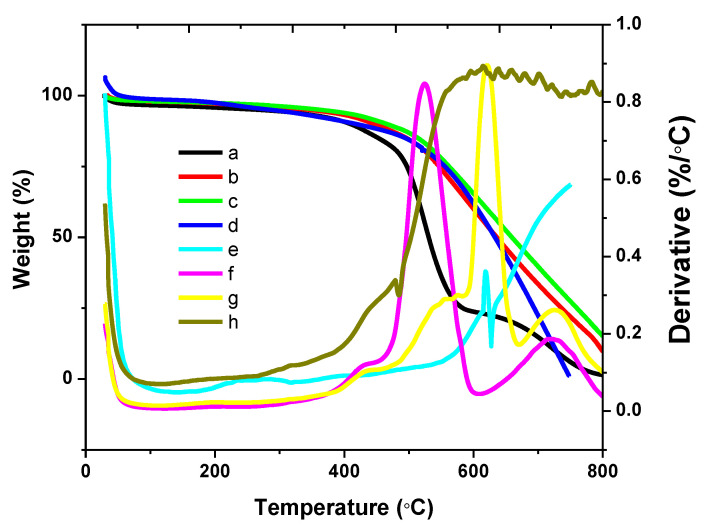
The hybrids temperature analysis: (**a**–**d**) percentage weight curve of $\mathrm{HGPPy}.{\mathrm{CB}}_{20\%}$ 1:3, $\mathrm{HGPPy}.{\mathrm{CB}}_{20\%}\;3:7$, $\mathrm{HGPPy}.{\mathrm{CB}}_{20\%}\;7:13$, $\mathrm{PPy}.{\mathrm{CB}}_{20\%}$ and (**e**–**h**) derivatives curve of $\mathrm{PPy}.{\mathrm{CB}}_{20\%}$, $\mathrm{HGPPy}.{\mathrm{CB}}_{20\%}$ 1:3, $\mathrm{HGPPy}.{\mathrm{CB}}_{20\%}\;3:7$, $\mathrm{HGPPy}.{\mathrm{CB}}_{20\%}\;7:13$.

The thermal stabilities of the individual material and the hybrids were succinctly investigated using the thermogravimetric analyzer (TGA), under nitrogen atmosphere. The temperature range of between 25 and 750 °C was chosen to study the individual material, while the hybrids were studied between 25 and 900 °C. As it is shown in Figure 4, the $\mathrm{PPy}.{\mathrm{CB}}_{20\%}$ experienced a linear decomposition profile from between 41.36 and 189.71 °C, due to the evaporation of moisture content. The degradation continues until 494.71 °C: a point where the final mass decomposition of the material was experienced until 750 °C. The $\mathrm{PPy}.{\mathrm{CB}}_{20\%}$ experienced a 56.36% total weight loss due to decomposition of the PPy backbone chain [48]. There is little or negligible mass decomposition for the Gr until 316.27 °C; a point where the Gr experienced a low, but steep degradation until 414.40 °C due to the decomposition of the amorphous carbon. A steady decomposition occurred at 574.9 ℃ until 750 ℃, due to Gr combustion. At 750 °C, ~13.62% total weight loss was observed for the Gr; this indicates the high thermal stability of Gr. By comparison, Figure 5 shows the thermal decomposition of the hybrids: $\mathrm{HGPPy}.{\mathrm{CB}}_{20\%}$ 1:3, $\mathrm{HGPPy}.{\mathrm{CB}}_{20\%}$ 3:7, and $\mathrm{HGPPy}.{\mathrm{CB}}_{20\%}$ 7:13. It is obvious from Figure 5 that the hybrid products and the $\mathrm{PPy}.{\mathrm{CB}}_{20\%}$ displayed similar thermal degradation patterns. However, the hybrids showed better thermal stability than the $\mathrm{PPy}.{\mathrm{CB}}_{20\%}$ from the temperature range of between 500 °C and upward. At this temperature range, the quantity of the hybrids residual weight increased as the weight of the Gr increased. 

**Figure 6:**
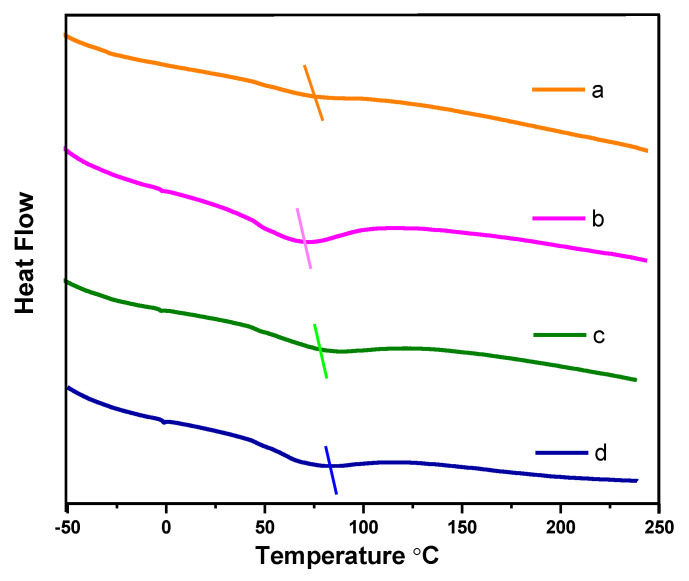
DSC thermograms for $\mathrm{PPy}.{\mathrm{CB}}_{20\%}$ and $\mathrm{HGPPy}.{\mathrm{CB}}_{20\%}$ (**a**) $\mathrm{PPy}.{\mathrm{CB}}_{20\%}$ (**b**) $\mathrm{HGPPy}.{\mathrm{CB}}_{20\%}$ 1:3 (**c**) $\mathrm{HGPPy}.{\mathrm{CB}}_{20\%}$ 3:7 (**d**) $\mathrm{HGPPy}.{\mathrm{CB}}_{20\%}$ 7:13.

Furthermore, the DSC analysis of the samples of the $\mathrm{PPy}.{\mathrm{CB}}_{20\%}$ and $\mathrm{HGPPy}.{\mathrm{CB}}_{20\%}$ were conducted in the temperature range of −65 °C to 240 °C. Glass transition can be used to discuss the materials conductivity because the interfacial interaction of fillers and polymer is a function of the glass transition [49,50]. The DSC investigation was carried out to determine the changes in the conductivity of the hybrids by observing the glass transition of the $\mathrm{PPy}.{\mathrm{CB}}_{20\%}$ as the weight fraction of the filler changes. As shown in Figure 6, the $\mathrm{PPy}.{\mathrm{CB}}_{20\%}$ shows an endothermic peak at 69.34 ℃, which is the glass transition of the $\mathrm{PPy}.{\mathrm{CB}}_{20\%}$. However, $\mathrm{HGPPy}.{\mathrm{CB}}_{20\%}$ 1:3 has an endothermic peak at 71.62 °C, while the peaks of $\mathrm{HGPPy}.{\mathrm{CB}}_{20\%}$ 3:7 and $\mathrm{HGPPy}.{\mathrm{CB}}_{20\%}$ 7:13 are around 82.11 °C and 83.17 °C; these peaks are the glass transition temperatures of the hybrids. Obviously, the glass transitions of the hybrid increase as the weight fraction of the filler content increases. The charge-transport mechanism of the hybrid can be explained by using the glass transition of the $\mathrm{PPy}.{\mathrm{CB}}_{20\%}$ and the $\mathrm{HGPPy}.{\mathrm{CB}}_{20\%}$. Glass transition of composite depends on the interface between the matrix and the filler: a strong interfacial interaction leads to an increase in glass transition. In contrast, a weak interface leads to a decrease in glass transition. The effect of glass transition on the electrical conductivity of polymer-composite is that electrical conductivity decreases with a decrease in glass transition and vice-versa. The DSC results displayed in Figure 6 show that the $\mathrm{HGPPy}.{\mathrm{CB}}_{20\%}$ glass transition increases as the weight fraction of the filler increases. In other words, as the weight fraction of the filler increases, the transport of electron increases due to an increase in the number of charge carriers. These results are in agreement with the literature [49,50,51].

### 5.3. Structural Characterization

The conductivity of polymer-composites in relation to crystallinity can be explained by percolation theory: this means that the relationship between crystallinity and conductivity of polymer-composite is not linear. As the crystallinity of polymer increases with respect to filler content and when a point beyond percolation threshold is reached, the conductivity becomes independent of the degree of the crystalline structure of the composite [52,53]. Consequently, the XRD investigation of the $\mathrm{HGPPy}.{\mathrm{CB}}_{20\%}$ is important to reveal their crystallinity in relation to electrical conductivity. 

**Figure 7:**
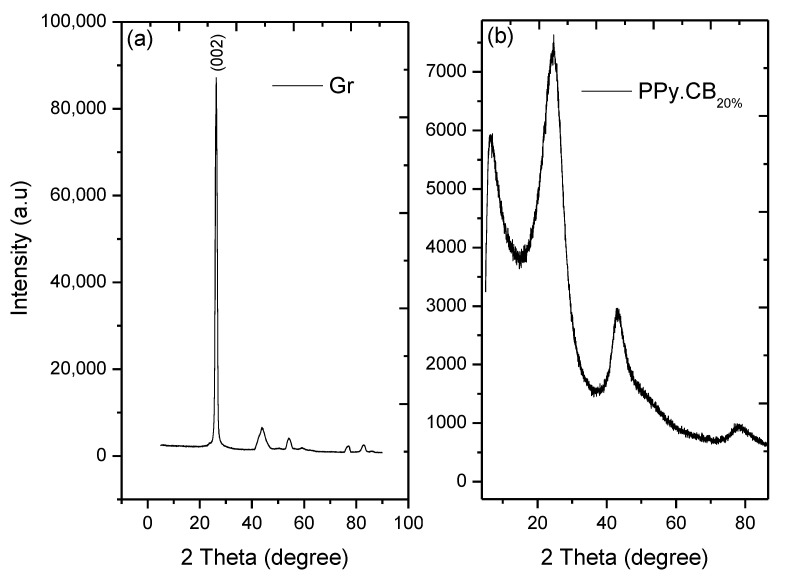
XRD pattern of (**a**) Gr (**b**) $\mathrm{PPy}.{\mathrm{CB}}_{20\%}.$

**Figure 8:**
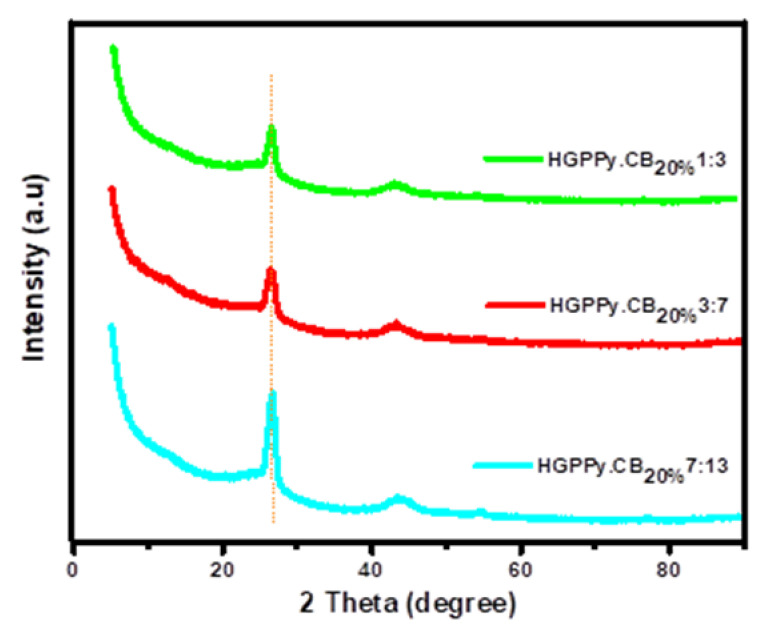
Comparison of the $\mathrm{HGPPy}.{\mathrm{CB}}_{20\%}$ structures.

The XRD patterns of the Gr, $\mathrm{PPy}.{\mathrm{CB}}_{20\%}$, and their hybrids are shown in Figure 7 and Figure 8. For the Gr, there exist high-intensity slim peak at 2θ=26.23° with four other small peaks, which are: 43.78°,  53.99°,  77.42°, and 82.10° corresponding to graphite-like structure [32]. The average interplanar distance and grain size of the Gr are 0.19 nm and 6.398 nm; and 97.40% crystallinity. The $\mathrm{PPy}.{\mathrm{CB}}_{20\%}$ structure showed three obvious peaks at 2θ=11.18 °, 24.6 °, and 41.2°. The broad peaks indicate CB and PPy, while the other peaks exemplified the presence of sulfonic acid in the material. The average crystal size and the d-spacing of the $\mathrm{PPy}.{\mathrm{CB}}_{20\%}$ were found to be 4.190 nm and 0.457 nm with ~57.51% crystallinity. However, as shown in Figure 8, by depositing Gr into the $\mathrm{PPy}.{\mathrm{CB}}_{20\%}$, its broad peak becomes very narrow or shrinks and shifts between 24.6° and 26.68°. The 11.18° peak disappeared while the third peak reduced. The clear indication of the interaction of the $\mathrm{HGPPy}.{\mathrm{CB}}_{20\%}$ is evident by the alteration of the $\mathrm{PPy}.{\mathrm{CB}}_{20\%}$ broad peak at 24.6° and the overlapping of the $\mathrm{PPy}.{\mathrm{CB}}_{20\%}$ at 26.68°. Moreover, the intensity of the $\mathrm{HGPPy}.{\mathrm{CB}}_{20\%}$ peak keeps increasing as the Gr content on the $\mathrm{PPy}.{\mathrm{CB}}_{20\%}$ increases. These results agree with the experimental work of Wang et al. [46] and Feng et al. [54]. 

### 5.4. HGPPy.CB_20%_ Electrical Conductivity

**Figure 9:**
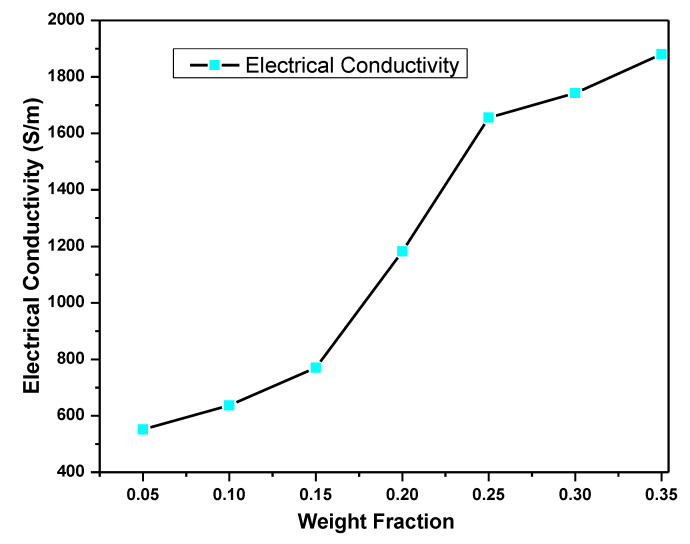
The experimental electrical conductivity of the $\mathrm{HGPPy}.{\mathrm{CB}}_{20\%}.$

The laboratory-measured electrical conductivity values of the as-received Gr and $\mathrm{PPy}.{\mathrm{CB}}_{20\%}$ are 2.04 × 10^−3^ S/m and 552 S/m, respectively. The electrical conductivity measurements for the $\mathrm{HGPPy}.{\mathrm{CB}}_{20\%}$ can be found in Figure 9. As shown in Figure 9, the electrical characteristics of the $\mathrm{HGPPy}.{\mathrm{CB}}_{20\%}$ changes as the weight fraction of the Gr changes. At Gr weight fraction of 0.15 wt.%, the hybrid begins continuous conduction, which connotes the percolation network formation. In other words, the percolation threshold of the hybrid occurred at 0.15 wt.%. At this point, there is a linear increment in the system’s electrical conductivity until 0.25 wt.%, when the hybrid begins to saturate. These results confirm the effectiveness and ability of Gr to improve polymers’ electrical conductivity at low or moderate quantity for diverse applications [32,55,56,57]. This, of course, can be attributed to the homogenous dispersion of Gr onto the $\mathrm{PPy}.{\mathrm{CB}}_{20\%}$ matrix and the ability of Gr to link the carrier transport through the van der Waals force and the π−π interactions exemplified by the materials [33,46]. 

### 5.5. Measurement and Model Comparison

**Figure 10:**
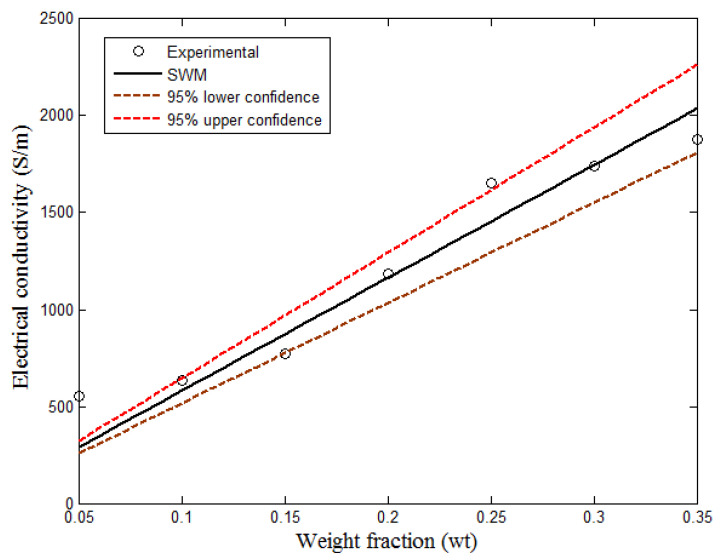
Comparison of experimental measurement with simplified Weber model (SWM).

Figure 10 shows the comparison of the experimental results with the Weber model. The Weber equation is, usually modeled with some parameters, such as orientation angle, a diameter of contact, filler length, filler diameter, and contact number. As shown in Figure 10, it is, vividly seen that Weber model does not give a percolation shape as expected. This is due to the assumptions, such as: contact number and the inadequacy of the model to consider the matrix effects and the percolation behavior of the hybrid. Therefore, the model may not be considered appropriate for the prediction of the electrical conductivity of polymer-composite [27,30]. However, the percolation threshold of the hybrid, as determined by the model on the logarithm scale, is almost equal to that of the experimental result. The equation of the linear graph of the simplified Weber model (SWM) is presented in Equation (14).

(14) $$y\left(x\right)=\beta_{1}\sigma_{f}x\;$$

where x is the weight fraction, $\sigma_{f}$ is the filler conductivity, y is the hybrid electrical conductivity, and $\beta_{1}$ is a value which described the effect of the various parameters in the Weber model. In addition, Equation (15) shows the value of $\beta_{1}$ with respect to filler conductivity and the weight fraction. More so, according to Taherian [31], the electrical conductivity of filler does not only show a power-law relationship, but it is also linear formation. Table 1 shows the calculated parameters and the accuracy of the prediction.

(15) $$y\left(x\right)=\beta_{1}\sigma_{f}x=2.8504\sigma_{f}x$$

**Figure 11:**
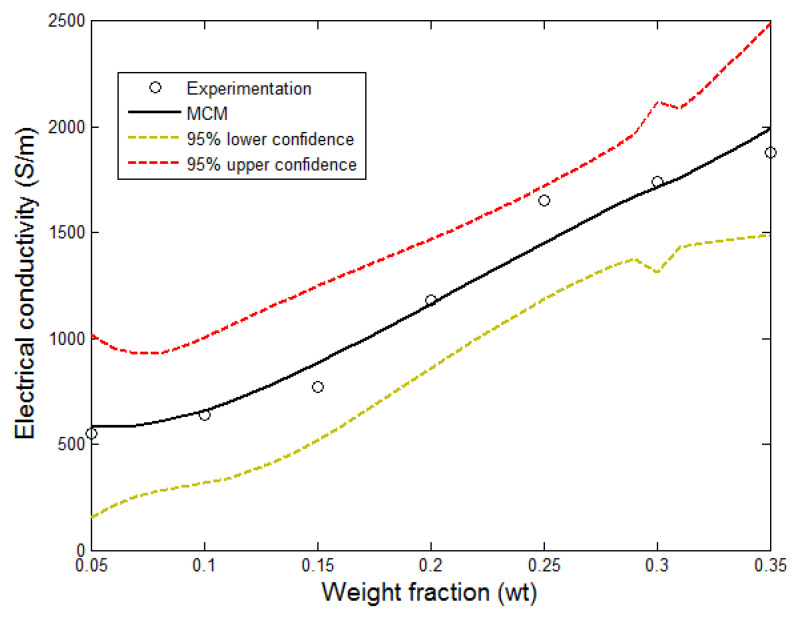
Comparison of experimental measurements with MCM.

For the Clingerman model, the value of the exponent t is found to be 1.11, from the experimental data. This exponential value controls the shape of the curve, while the other parameters, such as: orientation angle, surface interaction, and the aspect ratio, determine the growth of the filler on the matrix. The filler and matrix electrical conductivities are also important parameters in the model. As shown in Figure 11, the Clingerman model was able to trace the shape of the experimental curve of the $\mathrm{HGPPy}.{\mathrm{CB}}_{20\%}$. In order to reduce the complexity in the Clingerman model, Equation (8) was re-parameterized to obtain Equation (16).

(16) $$y\left(x\right)=\;\alpha_{1}+\;\alpha_{2}\sigma_{f}{\left(x-\;\alpha_{3}\right)}^{\alpha_{4}}$$

where x is the weight fraction, $\alpha_{1}$ is the sum of the matrix conductivity, the surface interaction effect, and the conductivity due to the aspect ratio, $\alpha_{2}$ is the constant D, $\alpha_{3}$ is the weight fraction, and $\alpha_{4}$ is the critical exponent. Table 2 provides the values of the calculated parameters of Equation (16), and the accuracy of the prediction.

The predictive model developed for the experimental data, is as presented in Equation (17).

(17) $$y\left(x\right)=\alpha_{1}+\alpha_{2}\sigma_{f}{\left(x-\alpha_{3}\right)}^{\alpha_{4}}=1720.8+3.8\sigma_{f}{\left(x-0.3\right)}^{1.11}\;$$

**Figure 12:**
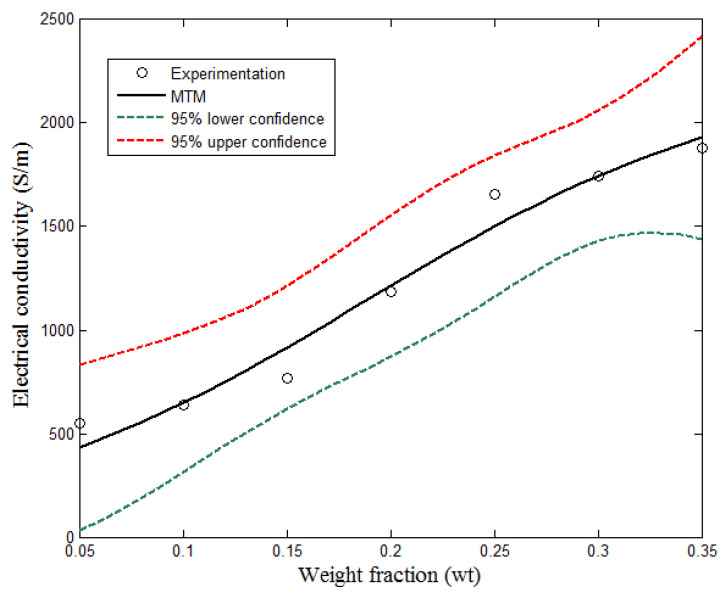
Comparison of experimental measurements with MTM.

Figure 12 compares the experimental results with Taherian model. The present study introduced a 5th parameter into the model for the purpose of a better predictive model. The factors considered in the model, include the aspect ratio, surface energy, orientation angle, and filler conductivity. The effect of the aspect ratio is inversely proportional to the percolation threshold and a large orientation angle leads to low electrical conductivity. The Taherian model shows a better analytical method of reproducing the experimental results. However, in order to obtain a suitable predictive model, Equation (12) is reduced to three parameters model, as shown in Equations (18) and (19).

(18) $$\sigma_{c}=\;\frac{a{}^{\circ}\sigma_{f}}{c{}^{\circ}+{\;\exp}^{-b{}^{\circ}\left(x-r\right)}}=\;\frac{\frac{a{}^{\circ}}{c{}^{\circ}}\sigma_{f}}{1\;+{\;\exp}^{-b{}^{\circ}x}\exp^{\frac{b{}^{\circ}r}{c{}^{\circ}}}}$$

Equation (18) is the three parameters model of the form provided by Equation (19).

(19) $$y\left(x\right)=\;\frac{\gamma_{1}\sigma_{f}}{1+e^{-\gamma_{2}\left(x-\;\gamma_{3}\right)}}\;$$

The results of the analytical predictive model of Equation (19) is given in Table 3.

The predictive model developed for the experimental data, according to Equation (19) is as presented in Equation (20).

(20) $$y\left(x\right)=\frac{\gamma_{1}\sigma_{f}}{1+e^{-\gamma_{2}\left(x-\gamma_{3}\right)}}=\frac{2278}{1+e^{-10.54\left(x-0.19\right)}}=\;\frac{1.099\;\sigma_{f}}{1+e^{-10.54\left(x-0.19\right)}}\;$$

From the results of these models, it is obvious that models can be adopted to measure the electrical conductivity of polymer-composites if the factors which determine the electrical conductivity of the composites are known. As stated before, these factors include aspect ratio, filler wettability, orientation angle, surface interaction, filler morphology, interfacial interaction, interfacial effect, filler and matrix conductivity, and some others. For accuracy of results, cost of experimentation, and efficient production of composites materials, modeling cannot be over-emphasized.

## 6. Conclusions

This study has demonstrated that graphene nanoplatelets can improve polymer electrical conductivity with further enhancements in its thermal stability, structural, and morphological patterns. The SSB process employed yielded a thermally stable and excellent $\mathrm{HGPPy}.{\mathrm{CB}}_{20\%}$, as a conductor. The measurements of the hybrids’ electrical conductivity show that the weight fraction of graphene nanoplatelets is central to modifying the intrinsic/extrinsic properties of the polymer. Weber, Clingerman, and Taherian models were used to describe the electrical conductivity measurements of the hybrids. The observation from the fitting of these models, to the experimental data is that, Clingerman and Taherian models can be, improved upon, in order to develop a robust, reliable, and efficient electrical conductivity model for polymer-composites. In addition, the Weber model could only describe the experimental data linearly. By considering the significant effects of graphene nanoplatelets on polypyrrole having a component of other carbon family, their hybrid is envisaged to be promising for energy storage applications. Further study will present a specialized electrical conductivity model that is, based on the existing equations for polymer-composites electrical conductivity prediction. 

## Figures and Tables

**Table 1:** The modified Weber model parameter.

Models	Parameter	Parameter Value	Standard Error	Per Unit Standard Error	R^2^	R^2^-Adj.
Weber	$\beta_{1}$	2.8504	0.1015	0.0453	0.923	0.907

**Table 2:** Modified Clingerman model (MCM) parameters.

Models	Parameters	Parameters Values	Standard Error	Per Unit Standard Error	R^2^	R^2^-Adj.
Clingerman	$\alpha_{1}$	1720.8	255.68	0.1486	0.964	0.927
	$\alpha_{2}$	3.8000	0.5413	0.1426		
	$\alpha_{3}$	0.3000	0.0480	0.1594		
	$\alpha_{4}$	1.1100	0.0132	0.0119		

**Table 3:** Modified Taherian predictive model (MTM) results.

Models	Parameters	Parameters Values	Standard Error	Per Unit Standard Error	R^2^	R^2^-Adj.
Taherian	$\gamma_{1}\sigma_{f}$	2278	408	0.1789	0.967	0.950
	$\gamma_{2}$	10.54	2.96	0.2810		
	$\gamma_{3}$	0.19	0.04	0.2270		

## Data Availability

Data available on request from the corresponding author (O.F).
